# Numerical simulation study on the optimization design of the crown shape of PDC drill bit

**DOI:** 10.1007/s13202-013-0091-9

**Published:** 2013-11-28

**Authors:** Pei Ju, Zhenquan Wang, Yinghu Zhai, Dongyu Su, Yunchi Zhang, Zhaohui Cao

**Affiliations:** MOE Key Laboratory of Petroleum Engineering in China, China University of Petroleum, Beijing, 102249 China

**Keywords:** PDC drill bit, Bit crown, Per revolution-specific rock-breaking work, Numerical simulation

## Abstract

The design of bit crown is an important part of polycrystalline diamond compact (PDC) bit design, although predecessors have done a lot of researches on the design principles of PDC bit crown, the study of the law about rock-breaking energy consumption according to different bit crown shape is not very systematic, and the mathematical model of design is over-simplified. In order to analyze the relation between rock-breaking energy consumption and bit crown shape quantificationally, the paper puts forward an idea to take “per revolution-specific rock-breaking work” as objective function, and analyzes the relationship between rock properties, inner cone angle, outer cone arc radius, and per revolution-specific rock-breaking work by means of explicit dynamic finite element method. Results show that the change law between per revolution-specific rock-breaking work and the radius of gyration is similar for rocks with different properties, it is beneficial to decrease rock-breaking energy consumption by decreasing inner cone angle or outer cone arc radius. Of course, we should also consider hydraulic structure and processing technology in the optimization design of PDC bit crown.

## Introduction

The crown shape of polycrystalline diamond compact (PDC) drill bit affects the stability, wear, bottom hole cleaning, and load distribution of the bit. Predecessors have done a lot of researches about the design of PDC bit crown, and proposed some design principles, such as equivolume, equal abrasion, and equipower (Shusheng et al. 1998; Rong et al. 2006; Zhigang and Gang 1994; Hibbs and Flom 1978; Glowka D 1985; Sinor et al. 1998). But they just got the conclusion that “the axial force and cutting force of the topper cutter is bigger than the both sides cutters” qualitatively (Han Laiju et al. 1992), and did not give the quantitative analysis. The wear of the cutters is mainly caused by the rock-breaking force and specific energy consumption, and the force that applied on the cutters in the different parts of the crown is different, so it is necessary to study the mechanical characteristic of cutters in different parts of the crown, and this research will has an important guiding significance in the optimization design of PDC drill bit’s cutting structure. This paper takes “per revolution-specific rock-breaking work” as objective function, evaluates the rock-breaking effect of different crown shapes, and provides the theoretical basis for the optimization design of PDC drill bit’s crown shape.

Generally, PDC bit’s crown shape consists of inner cone, cone apex, and outer cone. Inner cone takes effect on the stability and guiding of the bit; cone apex is the part which penetrates into the formation first, the design of the structure is related to the formation characteristics, the length of outer cone is determined by cutter density, and the outer cone affects the wear of the bit (Kerr 1988; Cerkovnik 1982). This paper analyzes the relation of inner cone angle and outer cone arc radius to per revolution-specific rock-breaking work, and lays the foundation for the optimization design of PDC drill bit’s crown shape.

## Per revolution-specific rock-breaking work

**Fig. 1:**
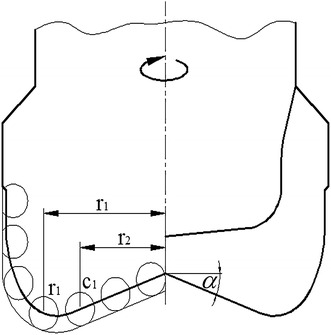
Sketch map of crown cutting unit

**Fig. 2:**
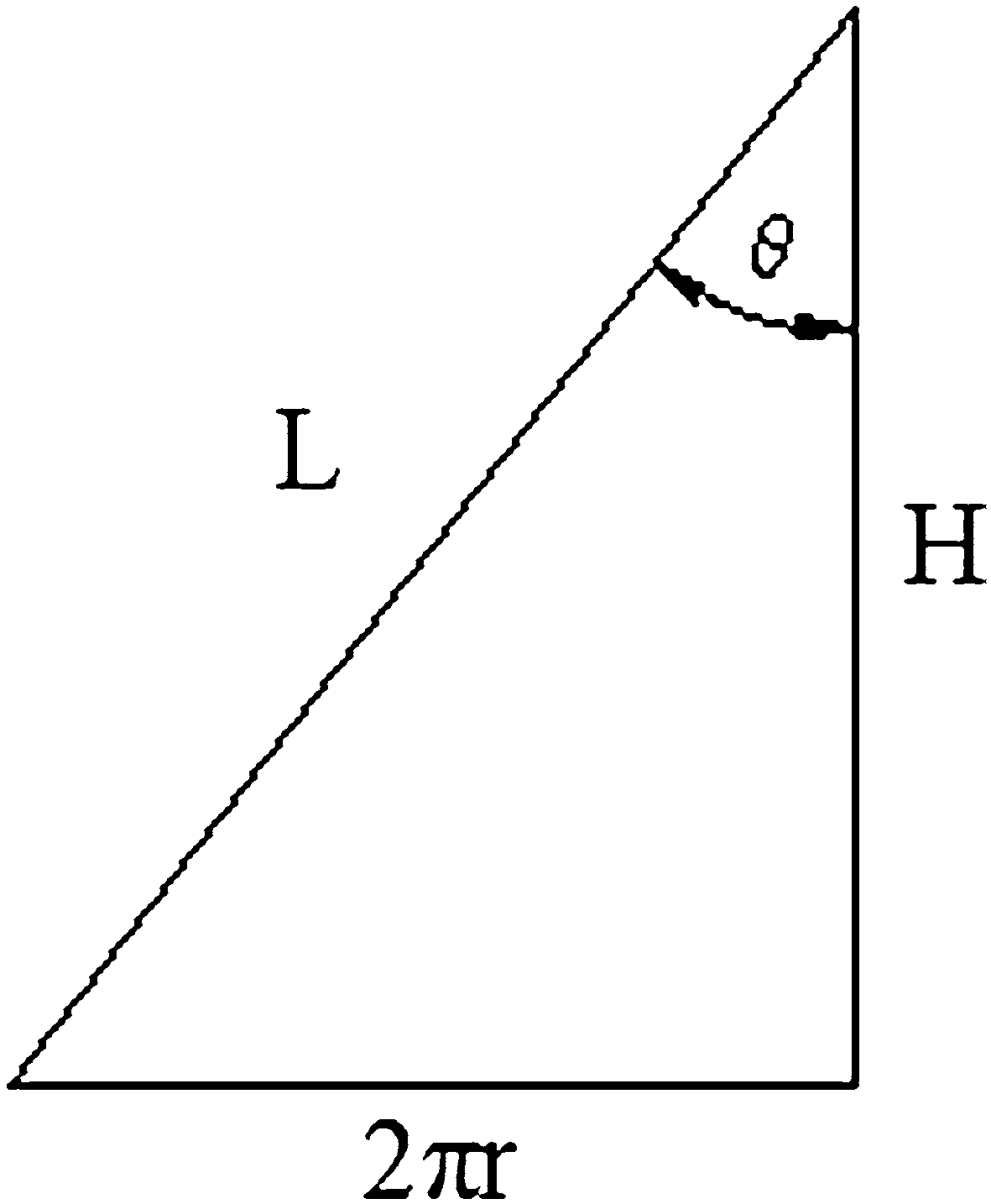
Relational graph between cutting displacement and depth

Because of the PDC drill bit’s self-sharpening, it penetrates into the formation under the drilling pressure, and shears formation with the action of torque, so the movement of the bit can be seen as a spiral progressing process. Presuming the distance between the cutter (C1) in the optional position of the bit crown and the bit axis is r1, the cutting angle of C1 is θ, per rotation displacement (spiral length) of C1 is *L*, and the depth of penetration is *H*. Stretching the spiral line along the direction parallels to the bit axis, there will be a right triangle relationship between *L* and *H*. Figure 1 is the sketch map of crown cutting unit, the inner cone angle is *a*, Fig. 2 is the schematic diagram of the relationship between *L* and *H*.

Presuming the work that the cutting unit requires to break per unit volume of rock in any position of the crown is *a*, so when this cutting unit rotates a circle round the bit, the work that this cutting unit does can be expressed as:

1 $$W = a \times (2\pi r) \times H$$

According to Fig. 2, can get the following displacement relation:

2 $$L = \sqrt {H^{2} + (2\pi r)^{2} } = \frac{2\pi r}{\sin \theta }$$

Based on the theory of doing work, there is the following relation:

3 $$W = F_{{\rm reslutant}} \frac{2\pi r}{\sin \theta }$$

Synthesizing formula 1 to 3:

4 $$a = \frac{{F_{\text{resultant}} }}{2\pi r\cos \theta }$$

Formula 4 can reflect the work that required to break per unit volume of rock in any position of the crown. The physical meaning of formula 4 reflects the work that the cutter requires to break per unit volume of rock in any position of the crown in the conditions of certain rock property, drilling technology, and drilling rate. The smaller of *a*, the less the rock-breaking specific energy consumption, and at the same time the rock-breaking efficiency will be higher. So, the parameter *a* can be used as the objective function for optimal design of PDC drill bit’s crown shape.

## Numerical simulation of rock-breaking process of the crown of PDC drill bit

In recent years, the finite element method has been used in the study of rock-breaking field because of its simplification and efficiency (Haghighi et al. 1985; Vyazmensky et al. 2007; Tang 1997; Yuchun et al. 2012; Yiji et al. 2009), this paper uses the explicit dynamic FEM (Ls-dyna) to simulate the rock-breaking process by PDC drill bit. Assuming the cutters are infinitesimal, the contact segments of the crown and rock can be seen as a holistic cutter. So, the rock-breaking process can be thought as the interaction part of the blade and rock, which under the force plumbed to the axis of the bit center, rotary cutting rock along the central axis of the bit.

### Material models

Considering the strain rate and damaging effect of the rock, this study uses plastic kinematic hardening model to simulate the rock’s material, which fixes the yield surface radius, and the center migrates along the direction of plastic strain (Ohno and Wang 1993; Agah-Tehrani et al. 1987). The yield condition is:

5 $$\phi = \frac{1}{2}\xi_{ij}^{2} - \frac{{\sigma_{y}^{2} }}{3} = 0$$

and $$\xi_{ij} = s_{ij} - \alpha_{ij} ,\,\,\sigma_{y} = \sigma_{0} + \beta E_{\rho } \varepsilon_{\text{eff}}^{P}$$

Considering the strain rate according to the Cowper–Symonds model (Gyliene and Ostasevicius 2011), the yield stress can be represented with the coefficient which related to the strain rate:

6 $$\sigma_{y} = \left[ {1 + \left( {\frac{{\dot{\varepsilon }}}{C}} \right)^{\frac{1}{p}} } \right]\left( {\sigma_{0} + \beta E_{p} \varepsilon_{\text{eff}}^{p} } \right)$$

7 $$\dot{\varepsilon } = \sqrt {\dot{\varepsilon }_{ij} \dot{\varepsilon }_{ij} }$$

The yield surface radius of *σ*_*y*_ equals to the sum of the initial yield strength *σ*_0_ and the increment $\beta E_{p} \varepsilon_{\text{eff}}^{p}$. The plastic hardening modulus can be represented as:

8 $$E_{\text{p}} = \frac{{E_{\text{t}} E}}{{E - E_{\text{t}} }}$$

The effective plastic strain is:

9 $$\varepsilon_{\text{eff}}^{p} = \int\limits_{0}^{t} {\left( {\frac{2}{3}\dot{\varepsilon }_{ij}^{p} \dot{\varepsilon }_{ij}^{p} } \right)}^{0.5} {\text{d}}t$$

10 $$\dot{\varepsilon }_{ij}^{p} = \dot{\varepsilon }_{ij} - \dot{\varepsilon }_{ij}^{e}$$

where *s*_*ij*_ deviatoric stress, *σ*_y_ yield stress, *σ*_0_ initial yield strength, *C*,*P* parameters of Cowper–Symonds strain rate, *ɛ*_eff_^*p*^ effective plastic strain, *E*_p_ plastic hardening modulus, *E* elasticity modulus, *E*_t_ shear modulus, $\dot{\varepsilon }_{ij}^{p}$ plastic strain rate, and $\dot{\varepsilon }_{ij}^{e}$ elastic strain rate.

This numerical simulation adopts the effective plastic strain rate failure criterion as the rock failure criterion. When the effective plastic strain reaches a given threshold, the element is damaged.

The main cutting part of PDC drill bit is polycrystalline diamond compacts, and its hardness and strength are far greater than the rock’s, so in order to improve calculation efficiency, this simulation treats the cutters as rigid materials, and defines the value of mass density, young’s modulus and poisson ratio of the rock. Table 1 gives the physical property parameters of cutter and rock, which are used in the simulation.

**Table 1:** Performance parameter of cutter and rock materials

	Density (g/cm^3^)	Young’s modulus (Mpa)	Tangent modulus (Mpa)	Yield stress (Mpa)	Poisson ratio
Cutter	7.83	2.1E4			0.3
Granite	2.64	5E4	0.55E4	4.0E4	0.28
Shale	2.64	2E4	0.2E4	5.1E4	0.15
Sandstone	2.65	4E4	0.4E4	1.4E4	0.25

### Finite element model

**Fig. 3:**
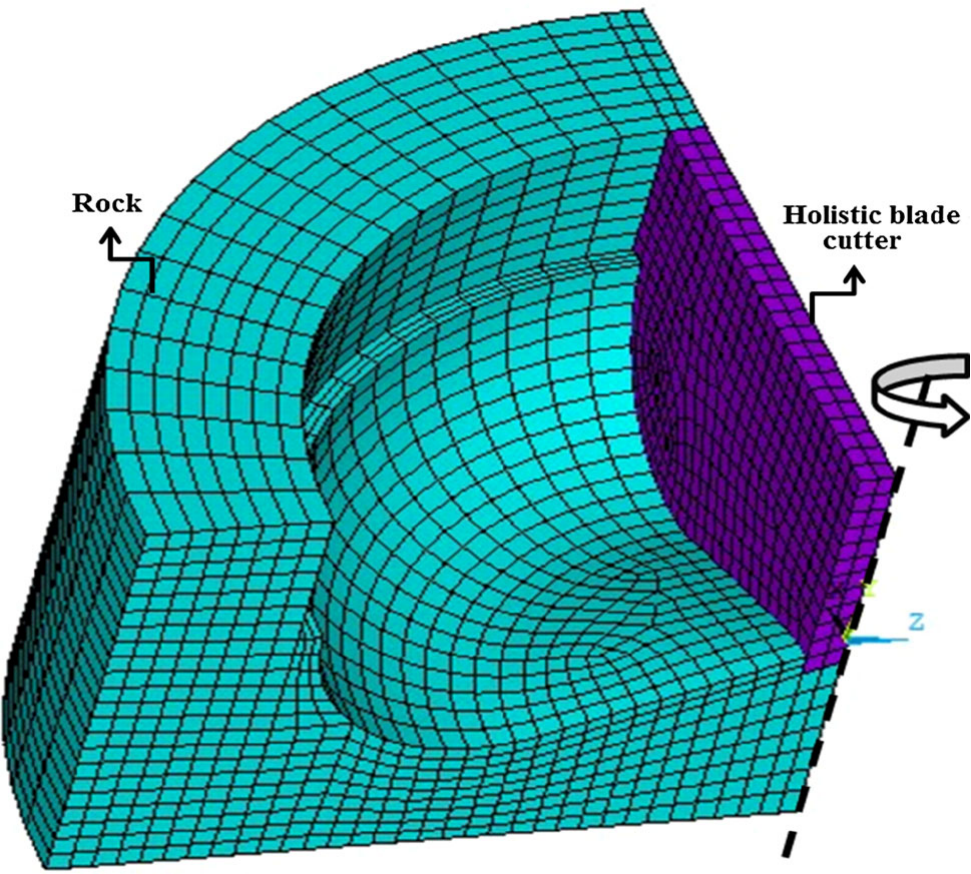
Simulated three-dimensional finite element model

As discussed above, this study treats the contact segment of the crown and rock as a holistic cutter, so this simulation adopts the finite element model that the entire blade rotary cuts the crown shape bottom hole, and uses the crown shape of “linear-circular-linear” as the blade shape. Figure 3 gives the three-dimensional model of the blade rotary cutting the rock.

In order to improve calculation efficiency, the finite element model takes a quarter of the actual formation model, the shape of the bottom hole coincides to the blade crown shape, the blade cutting face contacts with the rock at a certain angle, and the thickness of the blade is 2 mm. In order to simulate the real drilling situation, this model sets the frictional contact between the upper left side of the blade and the hole wall, and the blade does not cut the wall.

In this simulation, defines the mesh of the cutter and rock as eight nodes solid elements, and both of the cutter and rock are meshed with hexahedral sweeping. In order to avoid the effect of the boundaries wave reflection on the solution domain and simulate the infinite rock range, this simulation applies the nonreflecting boundary conditions on both outer circumferential surface and bottom surface of the rock. Also this simulation fixes all the degrees of freedom of the bottom surface of the rock, *x, y* translational degrees of freedom and *x, z* rotational degrees of freedom of the blade. The blade rotates around the central axis of the cylindrical formation at the speed of 30 r/min. In order to ensure the rest elements of the rock can still contact with the blade after the failure elements are deleted, this simulation defines erosion contact between the blade and rock. The calculation adopts the control of overall additional stiffness, and defines the stiffness coefficient no more than 5 % of the total internal energy. In order to avoid serious distortion of the grid during the calculation, this simulation calculates through Any Lagrangian–Eulerian (ALE) algorithm.

## Calculation results and analysis

**Fig. 4:**
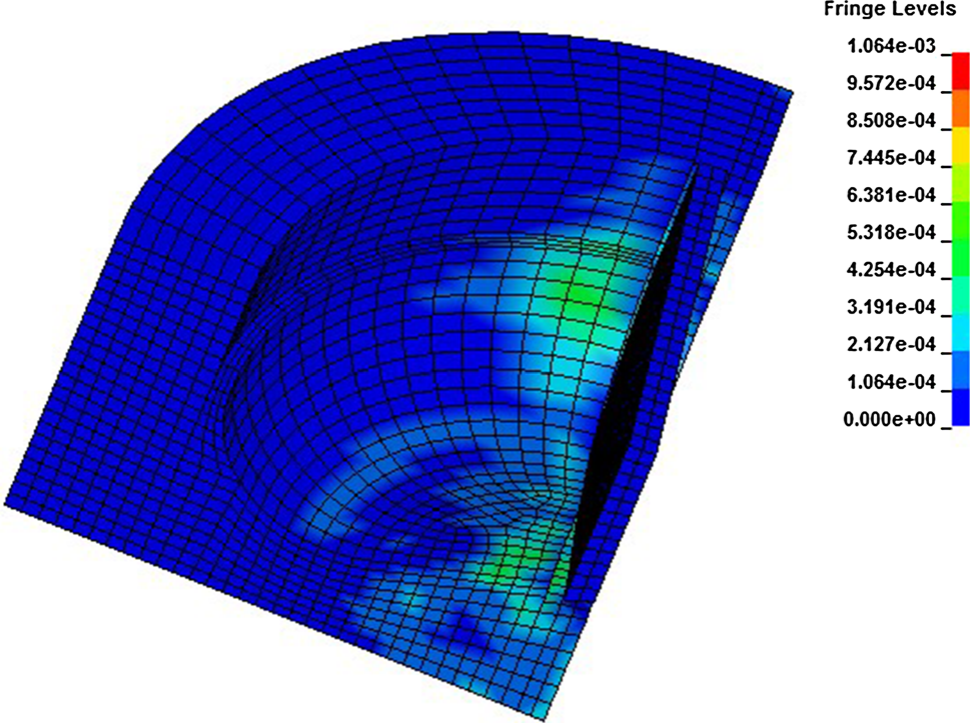
Rock Von Mises stress contour surface at some point

This study uses the above model and algorithm to simulate the rock-breaking process of different crown shape blade, and gets the resultant force at different positions of the crown through the post processing software “LS-PREPOST”, then substitutes the resultant force into formula 4, and finally gets the change rule between per revolution-specific rock-breaking work (*a*) and the radius of gyration (*r*). The simulated bit’s diameter is 8 1/2″. Table 2 shows the calculation parameters of the bit cutting structure. Figure 4 is the Von Mises stress contour surface of the rock at some point.

**Table 2:** Value of inner cone angle and outer cone arc radius in the simulation

Inner cone angle (°)	10	20	30
Outer cone arc radius (mm)	20	40	60

### Distribution law of per revolution-specific rock-breaking work along the radial direction

**Fig. 5:**
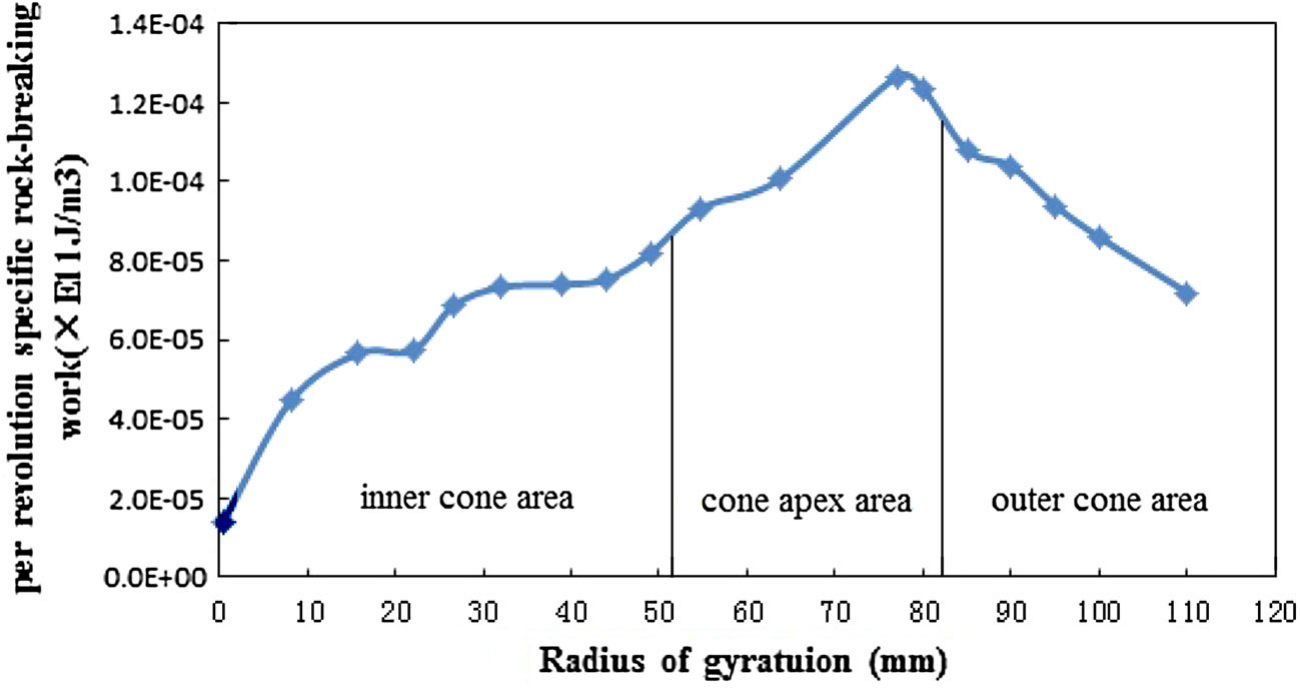
Change rule of per revolution-specific rock-breaking work with the increase of radius of gyration

When the blade rotates and cuts the rock, the bottom hole curvature at the top of the blade profile is the biggest, the rock’s free surface is less under the cutter of the cone apex, and adjacent cutters will generate a larger force on this rock, which will increase the compaction degree of this rock and lead the breaking of the rock at the cone apex more difficultly. Taking sandstone, for example, Fig. 5 shows the distribution regularity of the per revolution-specific rock-breaking work with the increase of rotation radius while inner cone angle is 40° and outer cone radius is 30 mm.

Figure 5 illustrates that, in the inner cone area, the cutting area of the rock is less near the center of the bit, per revolution-specific rock-breaking work *a* is smaller, and *a* increases slowly with the increase of the radius of gyration; in the cone apex area, *a* increases sharply with the increase of the radius of gyration, until to the bit nose position; in the outer cone area, *a* reduces gradually along with the increase of the radius of gyration. Thus, it can be seen that: rock stress is mainly concentrated on the drill bit nose area, extending from the bit nose area to the both sides of the nose, the rock stress diminishes gradually. This change rule is similar to the wear regularity of the bit (Han Laiju et al. 1992; Ai 2006), which proves the correctness of the above finite material model and computational model, and per revolution-specific rock-breaking work *a* can be used to reflect the rock breaking effect.

### Influence of rock property

**Fig. 6:**
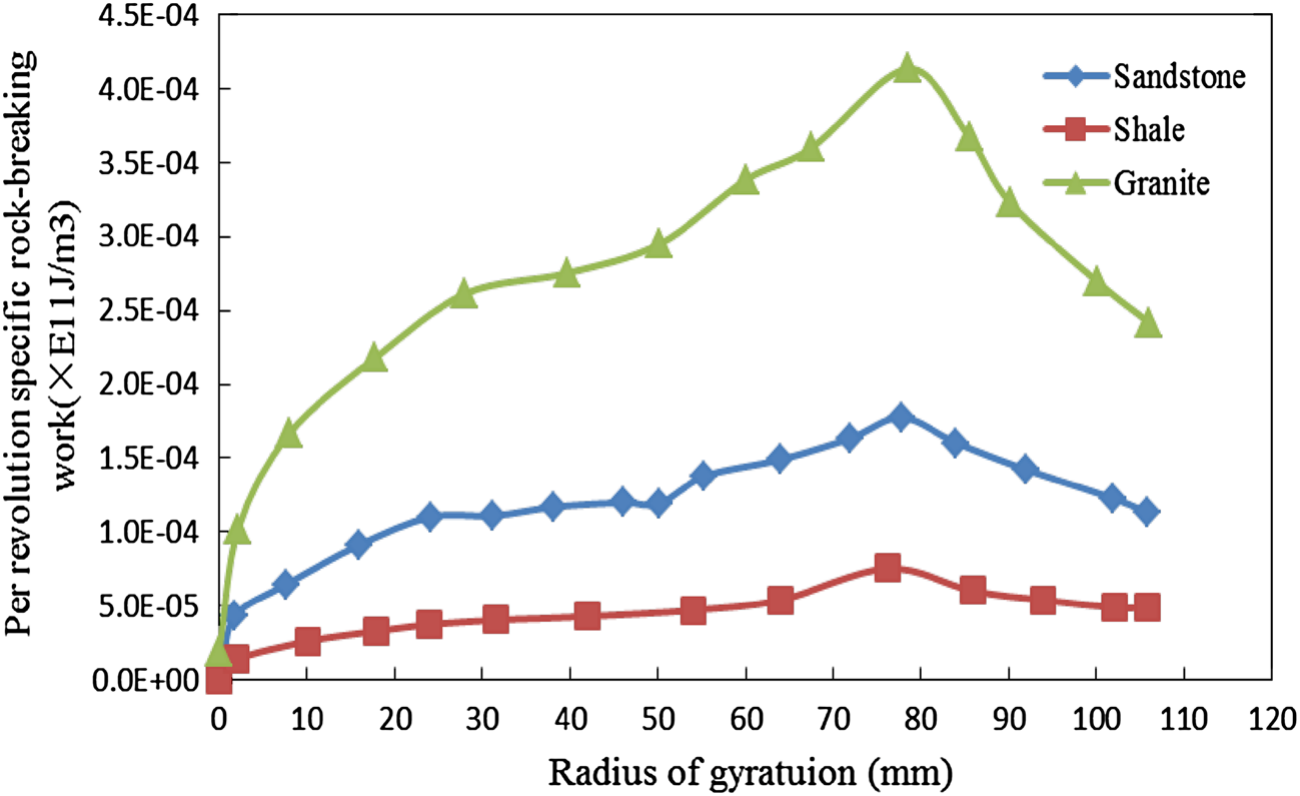
Influence of rock property on per revolution-specific rock-breaking work

Selecting the blade shape with inner cone angle 20° and outer cone radius 40 mm to cut granite, shale, and sandstone, respectively. Figure 6 shows the change law between per revolution-specific rock-breaking work and radius of gyration.

As seen in Fig. 6, when the rock property changed, the change rule between per revolution-specific rock-breaking work and radius of gyration are all similar with the same kind of crown shape. The greater the rock strength, the larger the work required to break per unit volume of rock at the same radius of gyration, also the steeper the changing trend of per revolution-specific rock-breaking work with the increase of radius of gyration.

### Influence of inner cone angle

**Fig. 7:**
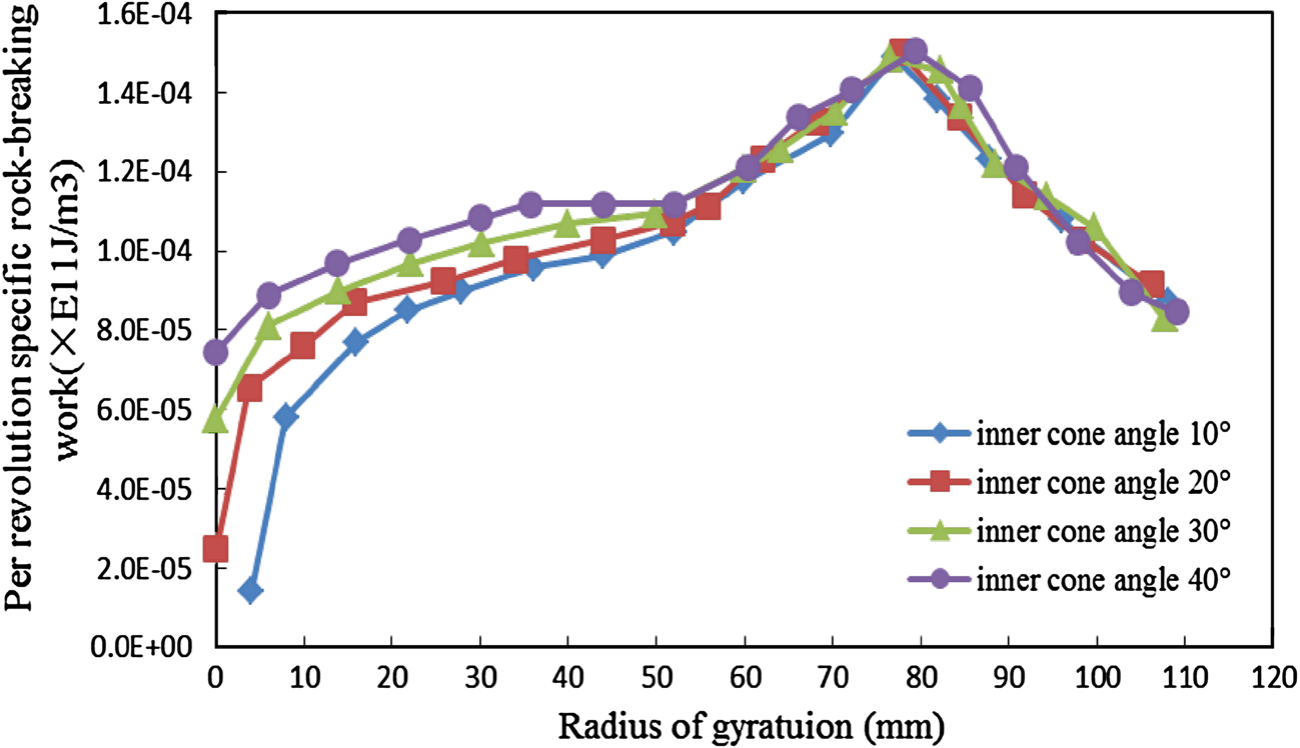
Influence of inner cone angle on per revolution-specific rock-breaking work

Because it has the same change rule of per revolution-specific rock-breaking work with the increase of radius of gyration for different types of rocks, this paper takes the sandstone as an example. Keeping the outer cone radius 30 mm unchanged, changes inner cone angle, and counts the change regulation of per revolution-specific rock-breaking work along with the increase of radius of gyration, as shown in Fig. 7.

From Fig. 7, it can be seen that: when the radius of gyration *r* < 40 *mm*, per revolution-specific rock-breaking work decreases with the increase of inner cone angle; when inner cone angle increases form 10° to 40°, per revolution-specific rock-breaking work decreases from 7.45 to 1.44 J/m^3^ in the central part of the bit, and the drop rate reached to 80 %; when the radius of gyration *r* > 40 *mm*, the change of per revolution-specific rock-breaking work of different inner cone angles is not obvious. This shows that, it is beneficial to reduce rock-breaking energy consumption and improve rock-breaking effect by increasing inner cone angle.

### Influence of outer cone arc radius

**Fig. 8:**
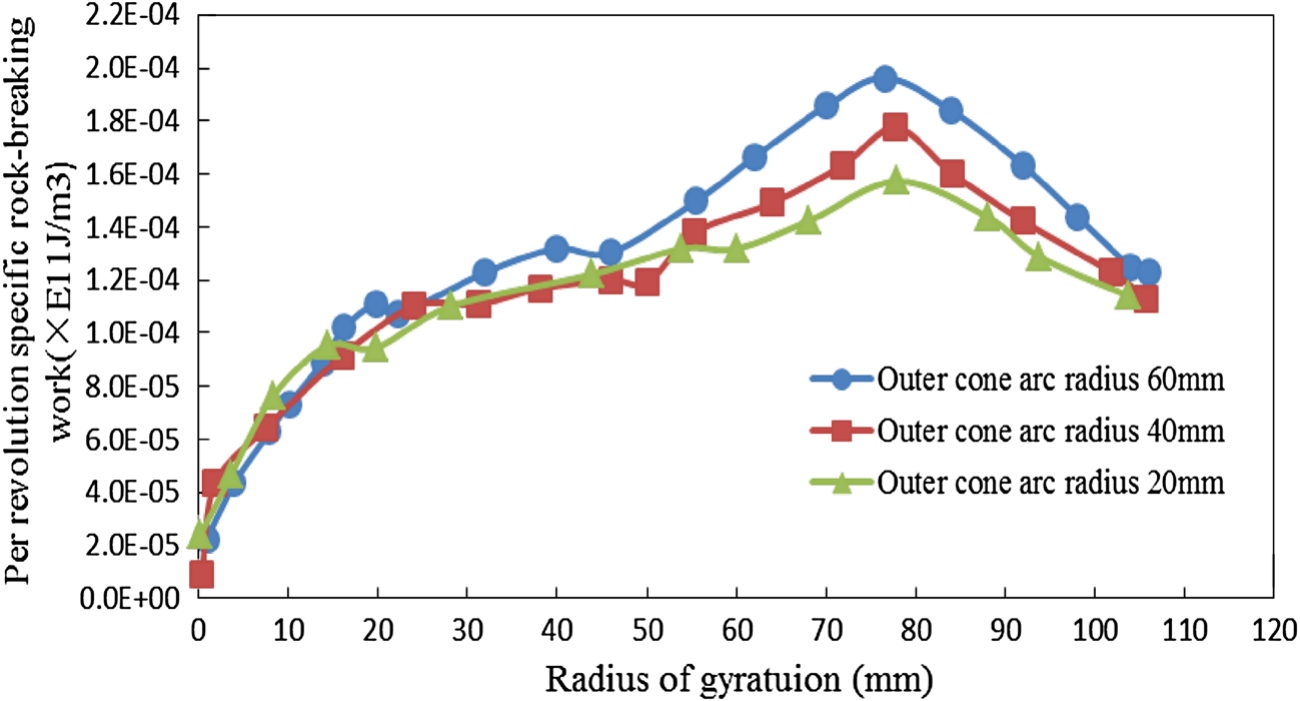
Influence of auto cone arc radius on per revolution-specific rock-breaking work

Also take sandstone for example, keeps the inner cone angle 20° unchanged, changing outer cone arc radius, and counts the change regulation of per revolution-specific rock-breaking work along with the increase of radius of gyration, as shown in Fig. 8.

From Fig. 8, it can be seen that per revolution-specific rock-breaking work is insensitive to outer cone arc radius in the area of inner cone. Extending outward from the cone apex area, per revolution-specific rock-breaking work increases with the increase of outer cone arc radius, and when the outer cone arc radius decreases from 60 to 20 mm, per revolution-specific rock-breaking work decreases by 19 %, so it is beneficial to reduce rock-breaking energy consumption by decreasing outer cone arc radius.

## Design of PDC bit for hardness-alternating formation

For the hardness-alternating formation, there are several phenomena: (1) part of the cutters will cut hard rock, these cutters will bear a large impact load, and the impact load can not only cause impact damage of cutters, but also reduce the stability of PDC bit; (2) DC bit will bear large lateral imbalance force, which lead the reverse circumnutation (whirling motion) of bit. The stress state of cutter change continuously, and the cutting area of each cutter is unstable, which will exacerbate the impact force consequently. Therefore, when design the PDC bit for hardness-alternating formation, it is not only need to improve the rock-breaking efficiency, but also should reduce the impact load on single cutter, improve the working stability, and prevent bit balling simultaneously.

**Fig. 9:**
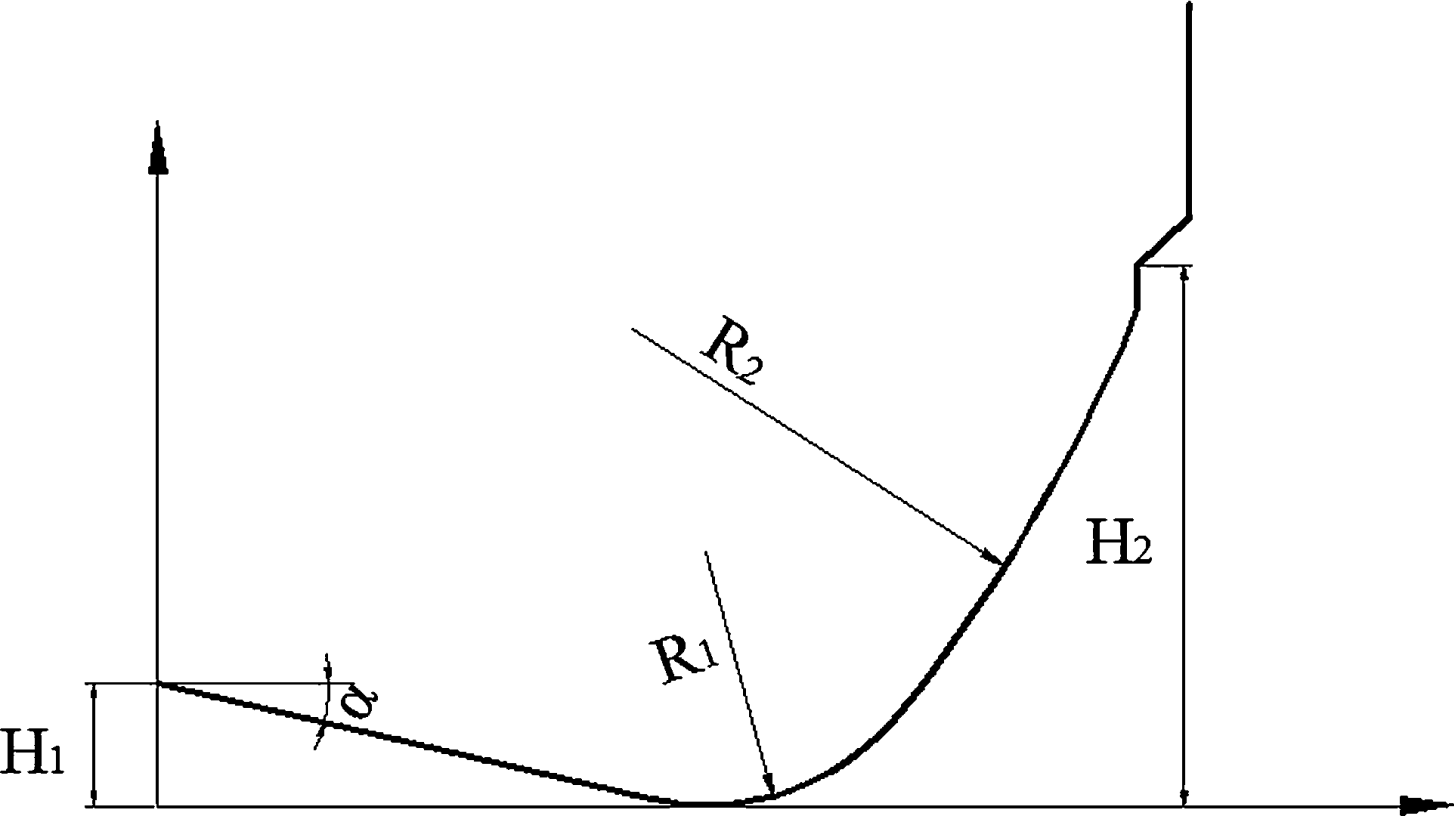
Crown profile designs diagram of the “straight line-arc-arc” type

According to the analysis above, the peak velocity of cutters on the exterior cone is larger than others, accordingly these cutters bear larger impact load; the smaller the inner cone angle, the less the rock-breaking energy consumption, however, the bit is more unstable. So, it is recommended to adopt “straight line-arc-arc” crown profile for hardness-alternating rocks. The double circular arc connection in the conic node can avoid the forming of sharp edge, and prevent early damage of the cutters in the conic node position. The inner cone angle should not be much smaller in order to improve the stability of the bit, the circular arc radius of the external cone (*R*_2_), and the exterior cone height (*H*_2_) should be larger so that it can increase the number of cutters and reduce impact load on cutters (as shown in Fig. 9), the specific parameters should be given based on rock characteristics.

**Fig. 10:**
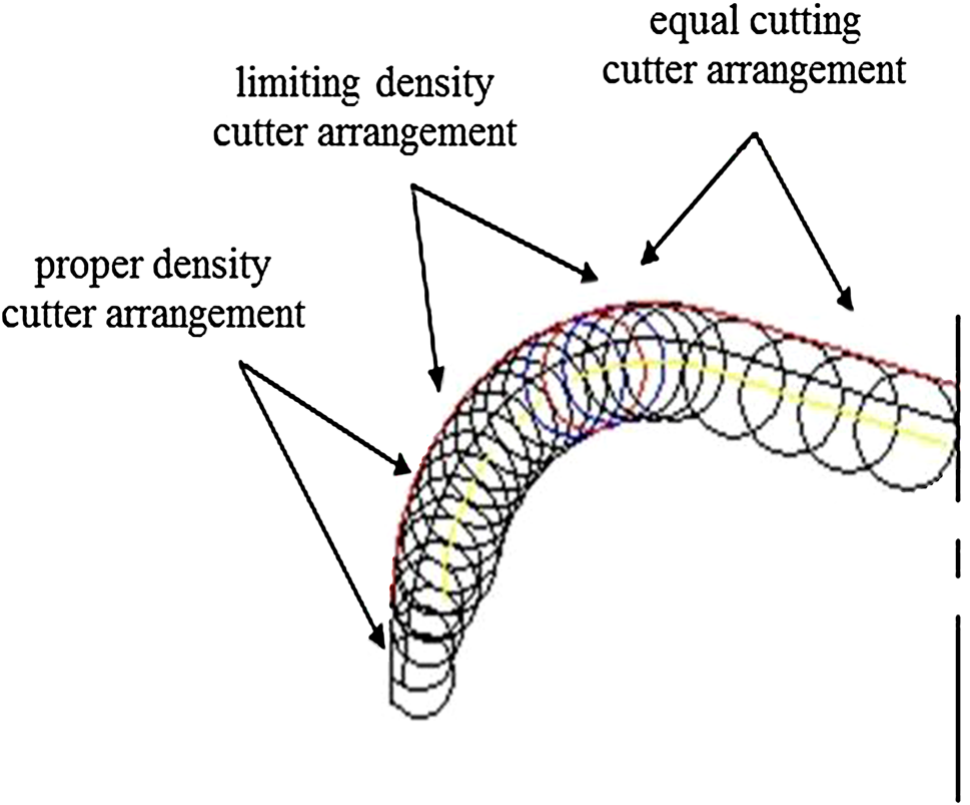
Maximizing the equivalent density of partial cutters in the serious wear place

**Fig. 11:**
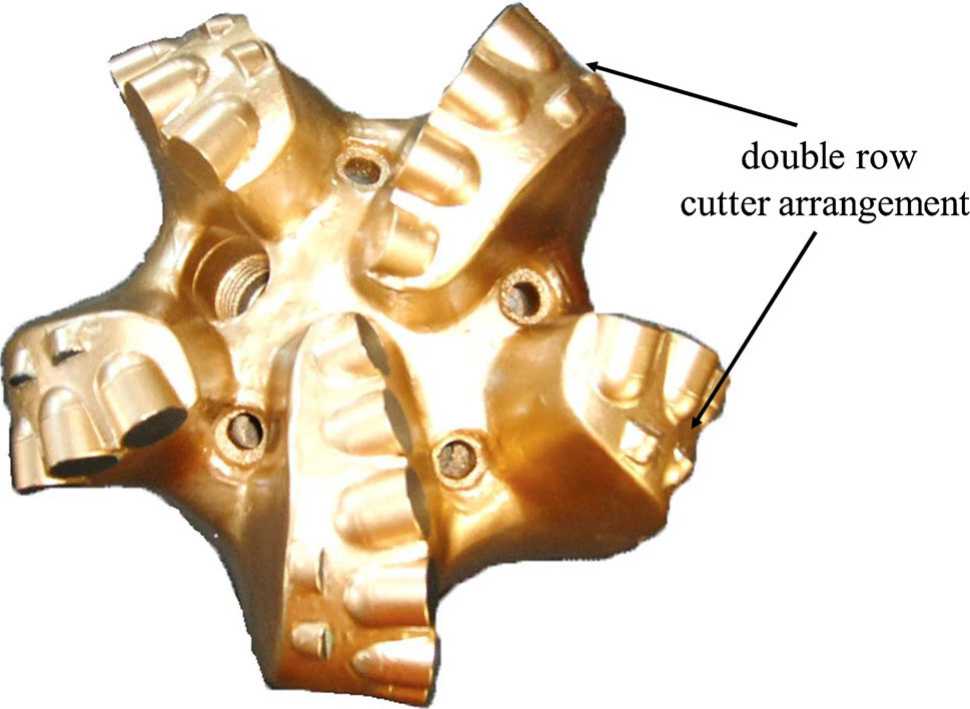
Double row cutter arrangement in serious wear place

**Fig. 12:**
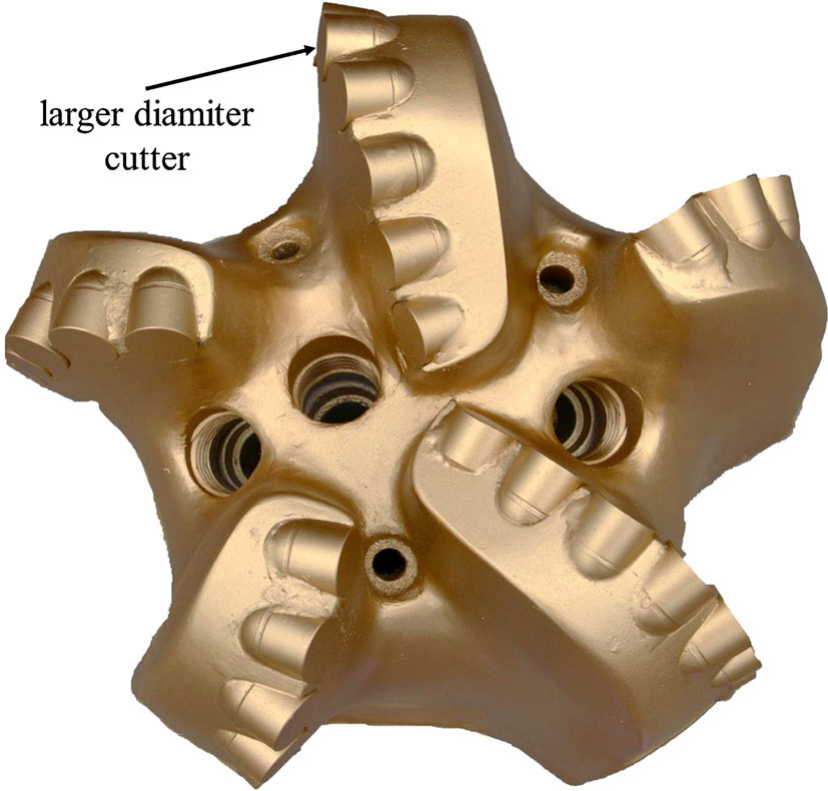
Adopting large diameter cutters in serious wear position

According to the characteristics of hardness-alternating formation, equal wear principle is adopted to design PDC bit. Through the statistical analysis of the bits using in oilfield, it can be known that the wear degree of cutters in different parts is consistent with the change law between per revolution-specific rock-breaking work and the radius of gyration. The wear extent of the cutters in interior cone is less, while the wear extent of the cutters at the cone apex is maximum. The thought of partial reinforcement is used, and the partial reinforcement measure is applied to the outer cone area near the outside of cone apex. The bit can be under “equal wear” condition by increasing the wear resistance of the exterior cone. The specific measures: (1) maximizing the equivalent density of partial cutters in the serious wear place (Fig. 10); (2) increasing the number of cutter in badly worn position, such as adopting double row cutter structure to restrict bit longitudinal vibration, improve bit’s impact resistance (Fig. 11) (3) improving the grade of cutters in badly worn position, adopting high strength, high wear resistant cutters in badly worn position to make the wear rate of all cutters consistent; (4) adopting large diameter cutters in serious wear position to increase the cutters’ wear resistance height (Fig. 12).

## Conclusion

1. The parameter *a* (per revolution-specific rock-breaking work) reflects that, under the conditions of certain rock property, drilling technology, and drilling rate, the work that the cutter requires to break per volume of rock in any position of the crown. Per revolution-specific rock-breaking work in any position of the crown acquired from finite element method is basically consistent with the wear law of PDC drill bit’s crown, this shows that it is reasonable to design the crown shape of PDC drill bit optimally based on the objective function of per revolution-specific rock-breaking work.
2. For different properties of rocks, the change law between per revolution-specific rock-breaking work and the radius of gyration is similar, the greater the rock strength, the larger the work that required to break per volume of rock at the same radius of gyration, also the steeper the changing trend of per revolution-specific rock-breaking work with the increase of radius of gyration.
3. The force on the inner cone area of shallow inner cone bit is smaller than deep inner cone bit. For the deep inner cone bit, the rock-breaking energy consumption is larger, and the cutters are easy to be damaged, while it is fairly stable for drilling.
4. The effect of outer cone arc radius on per revolution-specific rock-breaking work is mainly focus on the area of cone apex. Under the condition of certain other parameters, the bigger the outer cone arc radius, the greater the energy consumption of rock fragmentation.
5. The optimization design of the drill bit’s crown shape is not only related to the cutting structure, but also closely related to the hydraulic structure and processing technology. For the soft formation, whether the channel is unobstructed should be considered firstly. Then in this case, a larger value of the outer cone arc radius and a smaller value of the inner cone angel are suggested to choose. As for the hard formation, the wear of the cutters should be considered. So, a smaller value of the outer cone arc radius and a larger value of the inner cone angel are suggested to choose in order to reduce the rock-breaking energy consumption in the cone apex area.
